# PKC inhibitors reveal PKC isoforms involved in HIV latency reversal and immunomodulation

**DOI:** 10.1128/jvi.00500-26

**Published:** 2026-07-20

**Authors:** Thomas D. Zaikos, Victor Pham, Tessa C. Chou, Jose A. Moran, Shireen R. Turner, Brian H. Yu, Rami Hourani, Alok Ranjan, Jerome A. Zack, Paul A. Wender, Matthew D. Marsden

**Affiliations:** 1 Department of Microbiology and Molecular Genetics, School of Medicine, University of California 318244, Irvine, California, USA; 2 Department of Chemistry, Stanford University 198873, Stanford, California, USA; 3 Department of Microbiology, Immunology, and Molecular Genetics, University of California Los Angeles 158082, Los Angeles, California, USA; 4 Division of Hematology and Oncology, Department of Medicine, University of California Los Angeles 309947, Los Angeles, California, USA; 5 Department of Chemical and Systems Biology, Stanford University 196260, Stanford, California, USA; 6 Division of Infectious Diseases, Department of Medicine, School of Medicine, University of California 166662, Irvine, California, USA; University Hospital Tübingen, Tübingen, Germany

**Keywords:** HIV, PKC modulators, latency reversal, bryostatin-1, kick and kill

## Abstract

The reservoir of persistently latently infected cells is a major barrier to a cure for HIV infection. Protein kinase C (PKC) modulators can reverse HIV latency and could thus be useful “Kick” components in “Kick and Kill” approaches to a cure. However, PKC modulators also affect immune cell function, potentially limiting their clinical safety and utility. Here, using PKC isoform inhibitors in models of HIV latency and HIV-negative mononuclear cells, we determined the PKC isoforms involved in PKC modulator-mediated HIV latency reversal and immunomodulation (CD69 and CD4 expression, and inflammatory cytokine production). We found that inhibition of PKC α, β, γ, δ, or θ limited PKC-mediated HIV latency reversal to varying degrees. We also found that combinatorial PKC isoform inhibition significantly limited robust PKC modulator-mediated immune cell surface expression of CD69 and cytokine production. None of the pharmacologic PKC inhibitors affected PKC modulator-mediated downmodulation of T-cell surface CD4 expression. These results provide important insight into the isoforms involved in the various PKC modulator-mediated activities, including HIV latency reversal. Design of next-generation PKC modulators that are more selective for PKC α, β, and θ may allow for the partial functional decoupling of HIV latency reversal from immunomodulatory effects, and lead to safer and more effective PKC modulator-based latency-reversing regimens.

**IMPORTANCE:**

HIV persists in long-lived, latently infected cellular reservoirs, which prevents the cure of the infection using currently available antiretroviral therapy alone. The “Kick and Kill” strategy proposes the use of latency-reversing agents (LRAs) to induce viral reactivation leading to reservoir elimination. Protein kinase C (PKC) modulators are one of the most potent classes of LRAs and operate through the activation of several PKC isoforms. Here, we demonstrate the contribution of various PKC isoforms to PKC modulator-mediated HIV latency reversal and immunomodulation. We identified PKC α, β, and θ as the isoforms important for latency reversal, while other isoforms, especially broad PKC isoform activation, had greater relative effects on immune cell activation and cytokine release. Together, these results define the pathways required for PKC-mediated HIV latency reversal and other important immunomodulatory effects and will thus inform the development of next-generation isoform-selective PKC modulator LRAs.

## INTRODUCTION

Despite the efficacy of antiretroviral therapy, HIV persists in infected individuals within a reservoir of long-lived latently infected cells (1, 2). This persistent source of virus poses the major barrier to achieving a cure, necessitates life-long antiretroviral therapy, and makes infected individuals susceptible to the sequelae and complications of long-term infection and treatment (3, 4). Methods to eliminate the latent HIV reservoir have been proposed to effect a cure (5). One of the leading cure strategies is termed “Kick and Kill” and involves the use of HIV latency-reversing agents (LRAs) as the “Kick” component to induce reactivation of viral gene transcription and protein production, thus rendering infected cells susceptible to elimination through viral cytopathic effects or immune-mediated clearance, the “Kill” component (6). Therefore, to be successful, this approach requires LRAs to induce sufficient viral protein production in infected cells to maximize their clearance.

Several classes of LRAs have been proposed, developed, and investigated. Each LRA class leverages our understanding of HIV latency biology with the goal of inducing viral protein production to levels sufficient to induce reservoir clearance. Some, like histone deacetylase inhibitors and histone methyltransferase inhibitors, induce viral reactivation through chromatin remodeling, reducing the repressive chromatin structure around the viral promoter (7, 8). However, the majority of candidate LRAs affect signaling pathways and downstream transcription factors known to regulate HIV latency, such as cytokines and related compounds (9–14), benzotriazoles (15), disulfiram (16), toll-like receptor agonists (17, 18), second mitochondria-derived activator of caspases (SMAC) mimetics (19), and protein kinase C (PKC) modulators/agonists (20–22). Combinations of mechanistically distinct LRAs have also been tested (23, 24). Despite encouraging *in vitro* and *ex vivo* results, clinical translation of LRAs has thus far been unsuccessful due predominantly to limited *in vivo* efficacy (25–30). Nevertheless, ongoing work aims to address this obstacle to offer effective and tolerable LRA regimens.

PKC is a family of serine/threonine kinases involved in several cellular processes, including cell proliferation, differentiation, and apoptosis (31). There are three classes of PKC isoforms: conventional (α, β, and γ); novel (δ, ε, η, and θ); and atypical (ζ, ι, and λ), which differ in their structure, tissue distribution, and activation (32). PKC agonism has demonstrated potent HIV latency reversal activity in preclinical models (20, 23, 33–36). However, PKC modulators also cause a variety of immunomodulatory effects, including downregulation of surface CD4 on T cells and induction of pro-inflammatory cytokines (37–39). Therefore, identifying the roles of individual PKC isoforms in HIV latency reversal and PKC modulator-mediated immunomodulation could lead to the development of more targeted, more effective, and safer PKC modulator-derived LRA strategies.

Here, we report the specific PKC isoforms involved in PKC modulator-mediated HIV latency reversal and immunomodulation using cell line and primary cell models of HIV latency as well as peripheral blood mononuclear cells (PBMCs) from HIV-negative individuals. Elucidation of the specific isoforms involved in each of these processes will guide future efforts to develop novel, isoform-selective PKC modulators as LRAs with improved efficacy and tolerability.

## MATERIALS AND METHODS

### Virus production

Infectious supernatants were prepared by transfection of HIV-1 NL4-3 plasmid (40) into 293FT cells (Invitrogen). 293FT cells were cultured in Dulbecco’s modified Eagle medium (DMEM, Thermo Fisher) supplemented with 10% fetal bovine serum (FBS; Gibco) and 500 U/mL penicillin-streptomycin (Thermo Fisher). Transfections were performed using Lipofectamine 2000 (Invitrogen) according to the manufacturer’s instructions in Opti-MEM medium (Fisher Scientific) supplemented with 10% FBS. Cells were incubated for 1 day, at which time culture medium was replaced with DMEM with 10% FBS (without antibiotics), and the cells were incubated for another 2 days. Three days after transfection, infectious supernatants were collected, filtered through a 0.45 μm syringe filter (Millipore), aliquoted, and stored at –80°C.

### PBMC isolation

Whole blood from HIV-negative donors was obtained via the UCI Institute for Clinical and Translational Science (ICTS), and PBMCs were isolated by Ficoll-Paque PREMIUM (Fisher Scientific) density gradient centrifugation. Samples were collected by ICTS after UCI institutional review board approval and then provided in a de-identified fashion for the current study.

### PKC modulators

Bryostatin-1 and SUW133 were selected as representative PKC modulators for this study due to their extensive previous characterization, including similar single-digit nanomolar binding to PKC isoforms, their utilization in several *in vitro*, *ex vivo*, and *in vivo* studies, thus making them, especially SUW133, attractive candidate compounds for downstream clinical trials. The synthesis, characterization, measurement of PKC binding, and *in vitro* translocation of GFP-PKC isoform fusion proteins by the PKC modulators bryostatin-1 and SUW133 have been previously reported (20, 21, 41–43).

### PKC isoform inhibitor compounds

The following commercially available PKC isoform inhibitor compounds were used based on their reported biological activities against PKC isoforms: PKCε inhibitor V1-2/εV1-2 (MedChemExpress and Cayman Chemicals) (44, 45), ζ-Stat (MedChemExpress) (46, 47), PKCθ inhibitor (MedChemExpress and Selleck Chemicals) (48), PKCβ inhibitor 1 (MedChemExpress) (49, 50), and Go-6983 (MedChemExpress) (51, 52). Table 1 summarizes the specific inhibitor used and the complement of PKC isoforms inhibited by the compound at the concentrations used in this study. This panel of PKC isoform inhibitors represents compounds that affect the activity of all three categories of PKC isoforms: classical, novel, and atypical.

**TABLE 1:** Summary of pharmacologic PKC isoform inhibitors used^*a*^

		PKC isoforms inhibited								
PKC isoform inhibitor	Concentration used	Classical			Novel				Atypical	
		α	β	γ	δ	ε	η	θ	ζ	ι
ε-V1-2	25 μM									
ζ-Stat (NSC37044)	5 μM									
PKC θ inhibitor 2	1 μM									
PKC β inhibitor 1	1 μM									
Go 6983	50 nM									

^*a*^ PKC isoform inhibitors were purchased from MedChemExpress and used at the indicated concentrations, which are greater than the reported IC_50_ values for each of the affected PKC isoform (indicated by gray-shaded regions).

### J-Lat HIV latency reversal assay

Jurkat-Latency (J-Lat) cells were cultured and treated in RPMI 1640 medium supplemented with 10% FBS and 500 U/mL penicillin/streptomycin (R10 medium). J-Lat cells were plated in a 96-well round-bottom plate (50,000 cells/well) and treated with the indicated inhibitor compounds. For cells treated with PKC inhibitors, the PKC isoform inhibitor compound was added, plates were incubated at 37°C for 3 h, and PKC modulators were then added to the appropriate wells to the indicated final concentration. Cultures were incubated for 2 days total in a final culture volume of 200 μL/well. Cells were then pelleted, and HIV latency reversal was measured by flow cytometry. The following reagent was obtained through BEI Resources, NIAID, NIH: J-Lat Tat-GFP Cells (A2), ARP-9854. The following reagent was obtained through the NIH HIV Reagent Program, Division of AIDS, NIAID, NIH: J-Lat Full Length Cells (10.6), ARP-9849, contributed by Dr. Eric Verdin (53).

### Primary T_CM_ cell HIV latency reversal assay

Latently infected CD4^+^ T central memory-like (T_CM_) cells were generated as previously described, and the process is summarized in Fig. S1A (54). Briefly, naïve CD4^+^ T cells were isolated from HIV-negative donor PBMCs using a commercially available negative-selection magnetic sorting kit (STEMCELL Technologies) according to the manufacturer’s instructions (Day 0; Fig. S1B). Naïve CD4 T cells were cultured at 500,000 cells/mL in RPMI medium (GIBCO) supplemented with 10% FBS, 1% l-glutamine (Thermo Fisher), and 500 U/mL penicillin-streptomycin (T-cell culture medium). Naïve CD4 T cells were activated using colloidal polymeric nanomatrix conjugated to humanized CD3 and CD28 agonists (Miltenyi Biotec) in the presence of 1 μg/mL αIL-4 (Fisher Scientific), 2 μg/mL αIL-12 (Fisher Scientific), and 10 ng/mL TGF-β (Fisher Scientific) for 3 days. Cells were then washed (Day 3) and expanded for 4 days in culture medium supplemented with 30 IU/mL IL-2 (Fisher Scientific) and phenotyped on Day 7 before infection (Fig. S1C). Cells were infected with NL4-3 virus (Day 7) by spin inoculation (1,100 × *g* for 2 h at 37°C). Following spin infection, viral supernatant was removed, and cells were cultured in T-cell culture medium supplemented with IL-2. Three days post-infection (Day 10), culture infection rates were checked by flow cytometry (Fig. S1D) and cells were plated at 100,000 cells/well in 100 μL in 96-well round-bottom plates for 3 days to promote further spread of infection. Six days post-infection, infection rates (Day 13) were measured (Fig. S1E) and cells were uncrowded and cultured in standard tissue culture flasks in T-cell culture medium supplemented with IL-2 and antiretroviral compounds (1 μM raltegravir [Merck] and 0.5 μM nelfinavir mesylate [Fisher Scientific]), which was maintained and freshly prepared in all subsequent media changes and cultures. After 4 days (Day 17), CD4^+^ cells were isolated from infected cultures using a commercially available positive-selection magnetic sorting kit (Miltenyi Biotech; or STEMCELL Technologies) according to the manufacturer’s instructions with the modification that double the amount of reagents was used to increase purity and yield. Infection rate and CD4 sort efficiency were measured (Fig. S1F). All CD4 T cell sorts achieved >95% cell purity (average = 98.1%).

Isolated CD4^+^ T cells were then treated by plating between 200,000 and 300,000 cells/well/condition in a 96-well round-bottom plate with PKC inhibitor compounds for 1 h, followed by PKC modulators in a final volume of 200 μL in T-cell culture medium supplemented with IL-2 and antiretroviral compounds. Cells were treated for 48 h total, and HIV latency reversal was measured by flow cytometry. DMSO solvent and T-cell activation conditions were used in every experiment as negative and positive controls, respectively. T-cell activation was achieved by using colloidal polymeric nanomatrix conjugated to humanized CD3 and CD28 agonists (Miltenyi Biotec).

### Treatment of HIV-negative PBMCs for immunophenotyping assays

PBMCs from HIV-negative donors were obtained from the UCLA virology core following protocols approved by the UCLA Institutional Review Board. PBMCs were treated by plating 200,000 cells/well/condition in a 96-well round-bottom plate with the indicated compounds as described above (3 h PKC inhibitor treatment followed by PKC modulator treatment) in a final volume of 200 μL. Cells were treated for 48 h, at which point cells were pelleted, and culture supernatants were collected. Cell pellets were used for immunophenotyping by flow cytometry, and supernatants were used for cytokine and chemokine assays.

### Flow cytometry antibodies and staining

Antibodies raised against the following human antigens were used for flow cytometry: CD45 (clone 2D1, BioLegend, APC); CD3 (clone SK7, BioLegend, FITC or APC); CD3 (clone HIT3a, BioLegend, PerCP/Cyanine5.5); CD4 (clone OKT4, BioLegend, APC/Cyanine7 or PE); CD69 (clone FN50, BioLegend, Alexa Fluor 700); and HIV-1 core antigen (clone KC57, Beckman Coulter, RD1-conjugated). A commercially available panel was used to distinguish naïve and memory T cells (Human Naïve/Memory T Cell ID Panel, BioLegend #362201), which is made up of antibodies against CD3 (clone SK7, APC/Cyanine7), CD4 (clone SK3, PerCP/Cyanine5.5), CD45RA (clone HI100, FITC), and CD197/CCR7 (clone G043H7, APC). An antibody against CD45RO (clone UCHL1, BioLegend, Brilliant Violet 605) was also used for naïve and memory T-cell phenotyping. When indicated, a fixable viability dye (BioLegend) was used to measure cell viability.

For J-Lat HIV latency reversal experiments, cells were pelleted, washed with chilled PBS, and fixed in 2% paraformaldehyde (PFA) at 4°C overnight. HIV latency reversal was measured the following day based on GFP expression. Unstimulated/solvent-treated conditions were used as a negative control and for gating on positive events.

Flow cytometry was used to measure initial naïve CD4^+^ T cell sort purity and to confirm the generation of T_CM_ cells. To achieve this, 100,000–200,000 cells were pelleted, washed in chilled PBS, and incubated with fixable viability dye in PBS for 10–15 min at room temperature. Cells were washed with staining buffer (PBS with 2% FBS) and then incubated with antibodies against CD3 (clone SK7, APC/Cyanine7), CD4 (clone SK3, PerCP/Cyanine5.5), CD45RA (clone HI100, FITC), CD197/CCR7 (clone G043H7, APC), and CD45RO (clone UCHL1, Brilliant Violet 605) diluted in 50% human AB serum/PBS for 20 min at 4°C. Cells were washed with staining buffer and fixed in 2% PFA/PBS for 1 h at room temperature. Cells were pelleted, resuspended in staining buffer, and immediately analyzed.

To measure HIV latency reversal using the T_CM_ primary cell model, following 48-h treatment, cells were pelleted, washed in PBS, and resuspended in viability dye diluted in 100 μL PBS. Cells were stained with viability dye for 10 min at room temperature and then washed with staining buffer. Cells were then stained for surface CD3 by resuspending in antibody against CD3 (clone SK7, FITC) diluted in 100 μL of 50% human AB serum/PBS. Cells were stained for 20 min at 4°C before being washed in staining buffer. Cells were fixed and permeabilized by resuspending in 100 μL of a commercially available fixation/permeabilization solution (BD Biosciences #554714) for 1 h at room temperature. Cells were then washed twice in a commercially available permeabilization/wash buffer (BD Biosciences #554723) and then stained for intracellular HIV-1 gag/p24 protein by resuspending with antibody against HIV-1 gag/p24 (clone KC57, RD1-conjugated) diluted in permeabilization/wash buffer. Cells were stained for 1 h at 4°C, washed twice with staining buffer, resuspended in staining buffer, and immediately analyzed.

To measure cell surface expression of immune cell markers (CD45, CD3, CD4, and CD69), treated PBMCs were pelleted and stained with CD45 (clone 2D1, APC), CD3 (clone HIT3a, PerCP/Cy5.5), CD4 (clone OKT4, PE), and CD69 (clone FN50, Alexa Fluor 700) diluted in 50% human AB serum/PBS for 20 min at 4°C. Cells were washed in staining buffer, resuspended in 2% PFA, fixed overnight at 4°C, and analyzed the following day.

All samples were analyzed on a BD LSR Fortessa X20 flow cytometer with FACSDiva software (Becton Dickinson). Data were analyzed using FlowJo (Version 10.8.0; TreeStar Inc.).

### Measurement of culture supernatant cytokine and chemokine concentration

PBMCs (200,000 cells/well/condition) were plated and treated with the indicated PKC isoform inhibitors in a 96-well U-bottom plate for 3 h before the addition of SUW133 to a final concentration of 10 nM in a final culture volume of 200 μL. Cells were incubated for an additional 21 h (24 h total treatment), at which point cells and supernatants were collected. Supernatants were analyzed by the UCLA Immune Assessment Core using the human 38-plex Luminex cytokine/chemokine panel. Solvent/DMSO-treated culture supernatants were used as negative biological controls for each donor. Undetectable values were reported as the quantification limit for each cytokine/chemokine. Data for each cytokine/chemokine in each of the four independent biologic replicates were normalized as the fold increase compared to solvent/DMSO treatment.

### Statistics

Statistical analyses were performed using GraphPad Prism (version 10.4.2). The statistical analysis used is indicated in each figure legend. *P* < 0.05 was considered statistically significant.

## RESULTS

**Fig 1:**
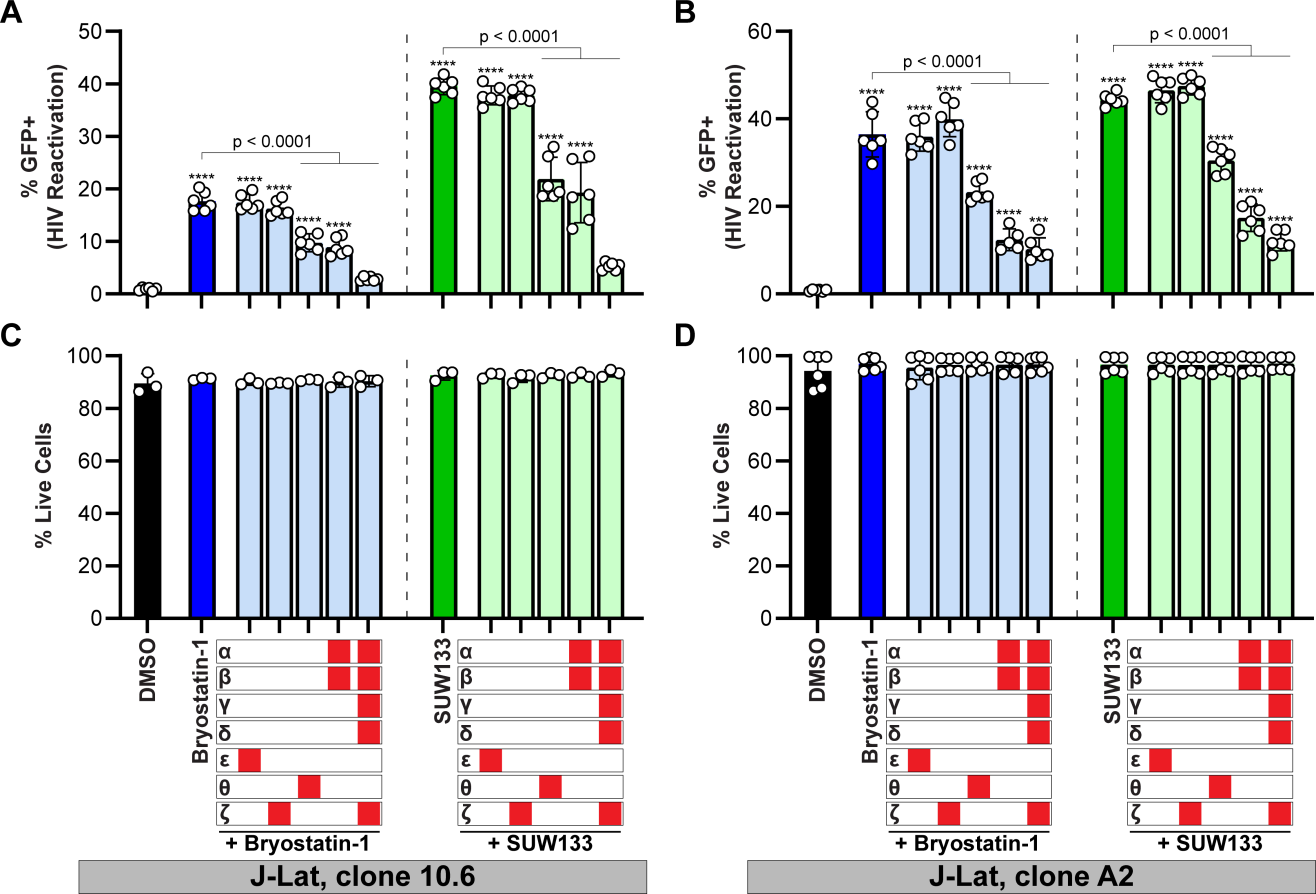
Effects of PKC isoform inhibition on PKC modulator-mediated HIV latency reversal in J-Lat cell lines. Cells were pre-incubated with commercially available PKC isoform inhibitors for 3 h and then stimulated with either 20 nM of bryostatin-1 or 10 nM of SUW133 for an additional 45 h (48 h total treatment). HIV latency reversal from (**A**) J-Lat (clone 10.6) and (**B**) J-Lat (clone A2) was measured by flow cytometry based on the percent of GFP+ cells. (**C and D**) Percent live cells in each culture condition were determined by flow cytometry. The mean and standard deviation are plotted for each condition. *N* = 6 (A), 5–6 (B and D), and 3 (C) experimental replicates. Statistical significance was determined using an ordinary one-way ANOVA with a Tukey post hoc test. Conditions that are significantly different from DMSO/solvent control are indicated with asterisks immediately above the bar. Statistically significant inhibition of PKC modulator-mediated HIV latency reversal is indicated by the grouped *P* value (***P < 0.001 and ****P < 0.0001).

We first sought to identify the PKC isoforms involved in PKC modulator-mediated HIV latency reversal in the Jurkat-derived HIV latency cell line model system (clones: J-Lat 10.6 and J-Lat A2). To accomplish this, we used commercially available pharmacologic PKC isoform inhibitors in combination with PKC modulators in HIV latency reversal assays. We chose bryostatin-1 and SUW133, a more potent and better tolerated bryostatin-1 analog, as representative PKC modulators, which have already demonstrated significant HIV latency reversal capabilities *in vitro*, *ex vivo*, and in HIV-infected humanized mice (20, 21, 55). Both J-Lat clones demonstrated minimal background/spontaneous reactivation with DMSO/solvent and significant HIV latency reversal with bryostatin-1 (Fig. 1A and B, 20- to 44-fold, *P* < 0.0001) and SUW133 (Fig. 1A and B, 44- to 54-fold, *P* < 0.0001). In both J-Lat clones, inhibition of PKC ε and ζ did not significantly affect bryostatin-1- or SUW133-mediated latency reversal. However, inhibition of PKC θ resulted in a significant reduction in bryostatin-1- (45–48% reduction, *P* < 0.0001) and SUW133-mediated (31–45% reduction, *P* < 0.0001) HIV latency reversal in both J-Lat clones (Fig. 1A and B). Inhibition of PKC α and β or broad PKC isoform (α, β, γ, and δ) inhibition led to a significant reduction in bryostatin-1-mediated (50–84% reduction, *P* < 0.0001) and SUW133-mediated (51–87% reduction, *P* < 0.0001) latency reversal in J-Lat 10.6 cells (Fig. 1A). Similar results were observed in J-Lat A2 cells (Fig. 1B; bryostatin-1 = 72–77% reduction, *P* < 0.0001; SUW133 = 61–73% reduction, *P* < 0.0001). Importantly, these results were not confounded by toxicity as each condition demonstrated excellent and similar cell viability (Fig. 1C and D).

**Fig 2:**
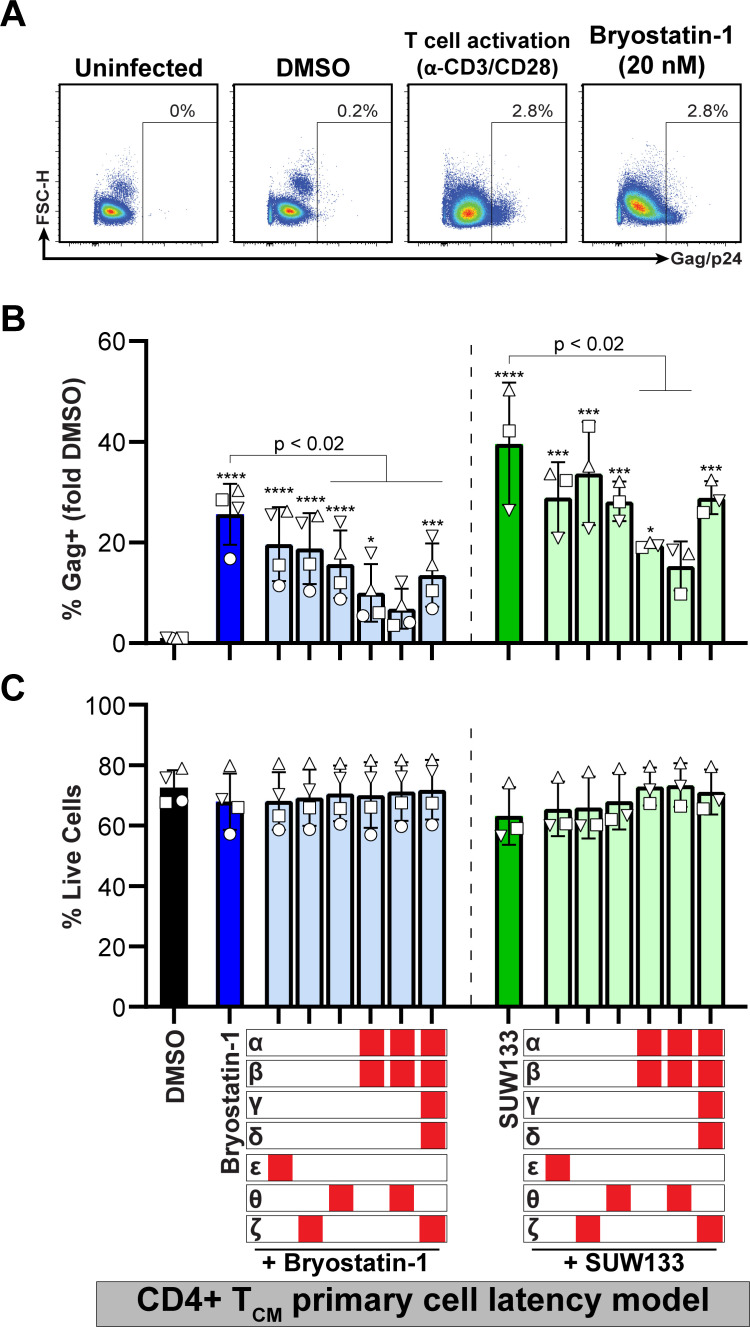
Effect of PKC isoform inhibition on PKC modulator-mediated HIV latency reversal in a primary cell HIV latency model. Cells were pre-incubated with commercially available PKC isoform inhibitors for 1 h and then stimulated with either 20 nM of bryostatin-1 or 10 nM of SUW133 for an additional 47 h (48 h total treatment) prior to measuring HIV latency reversal by flow cytometry. (**A**) Representative flow cytometric plots measuring HIV latency reversal based on HIV gag/p24. (**B**) Percent intracellular Gag^+^ cells after 48-h treatment (HIV latency reversal) normalized for each donor to the DMSO/solvent negative control. (**C**) Percent live cells at the end of the treatment in each culture condition were determined by flow cytometry. The mean and standard deviation are plotted for each condition. *N* = 3–4 unique HIV-negative donors. Statistical significance was determined using an ordinary one-way ANOVA with a Tukey post hoc test. Conditions that are significantly different from DMSO/solvent control are indicated with asterisks immediately above the bar. Statistically significant inhibition of PKC modulator-mediated HIV latency reversal is indicated by the grouped *P* value (*P < 0.05, ***P < 0.001, and ****P < 0.0001).

Given the artificial and homogenous nature of immortalized cell line models of HIV latency, we next wanted to test the ability of PKC isoform inhibitors to reduce PKC modulator-mediated latency reversal in a primary cell model of HIV. To accomplish this, we performed latency reversal assays using combinations of PKC isoform inhibitors and PKC modulators, as above, in a well-characterized and commonly used primary CD4^+^ T_CM_ cell model of HIV latency (Fig. S1) (15, 54, 56, 57). Background/spontaneous reactivation in this model was minimal (<1%) and we saw robust viral latency reversal in this system with T-cell activation and bryostatin-1 treatment (Fig. 2A). By itself, bryostatin-1 induced significant latency reversal (Fig. 2B; 29-fold over DMSO; *P* < 0.0001). Inhibition of PKC ε and ζ did not significantly affect bryostatin-1-mediated latency reversal (Fig. 2B). However, bryostatin-1-mediated reactivation was significantly reduced by inhibition of PKC θ (37% reduction, *P* = 0.016), PKC α and β (60% reduction, *P* = 0.0002), combined PKC α, β, and θ (73% reduction, *P* < 0.0001), and broad/pan PKC isoform inhibition (43% reduction, *P* = 0.0034) (Fig. 2B). Interestingly, with combined PKC α, β, and θ inhibition, the degree of bryostatin-1-mediated viral reactivation was suppressed to a degree that was statistically similar to that of solvent/background (Fig. 2B; *P* = 0.26).

SUW133-mediated latency reversal was also significantly above DMSO/solvent (Fig. 2B; 40-fold; *P* < 0.0001), although not significantly above latency reversal induced by bryostatin-1 (*P* = 0.20; two-tailed *t*-test). Inhibition of PKC ε and ζ did not significantly affect SUW133-mediated latency reversal (Fig. 2B). Like bryostatin-1, SUW133-mediated reactivation was also significantly reduced by inhibition of PKC α and β (51% reduction, *P* = 0.013) and PKC α, β, and θ combination (61% reduction, *P* = 0.0027). Unlike with bryostatin-1, we did not observe significant suppression of SUW133-mediated latency reversal with PKC θ (*P* = 0.30) or broad/pan PKC isoform (*P* = 0.37) inhibition (Fig. 2B). However, similar to bryostatin-1, so potent was the effect of PKC α, β, and θ inhibition on SUW133-mediated latency reversal that this combination did not result in latency reversal above that of solvent/background (Fig. 2B; *P* = 0.12; two-tailed *t*-test). These effects were also not confounded by treatment toxicity as each condition demonstrated excellent and similar cell viability (Fig. 2C).

**Fig 3:**
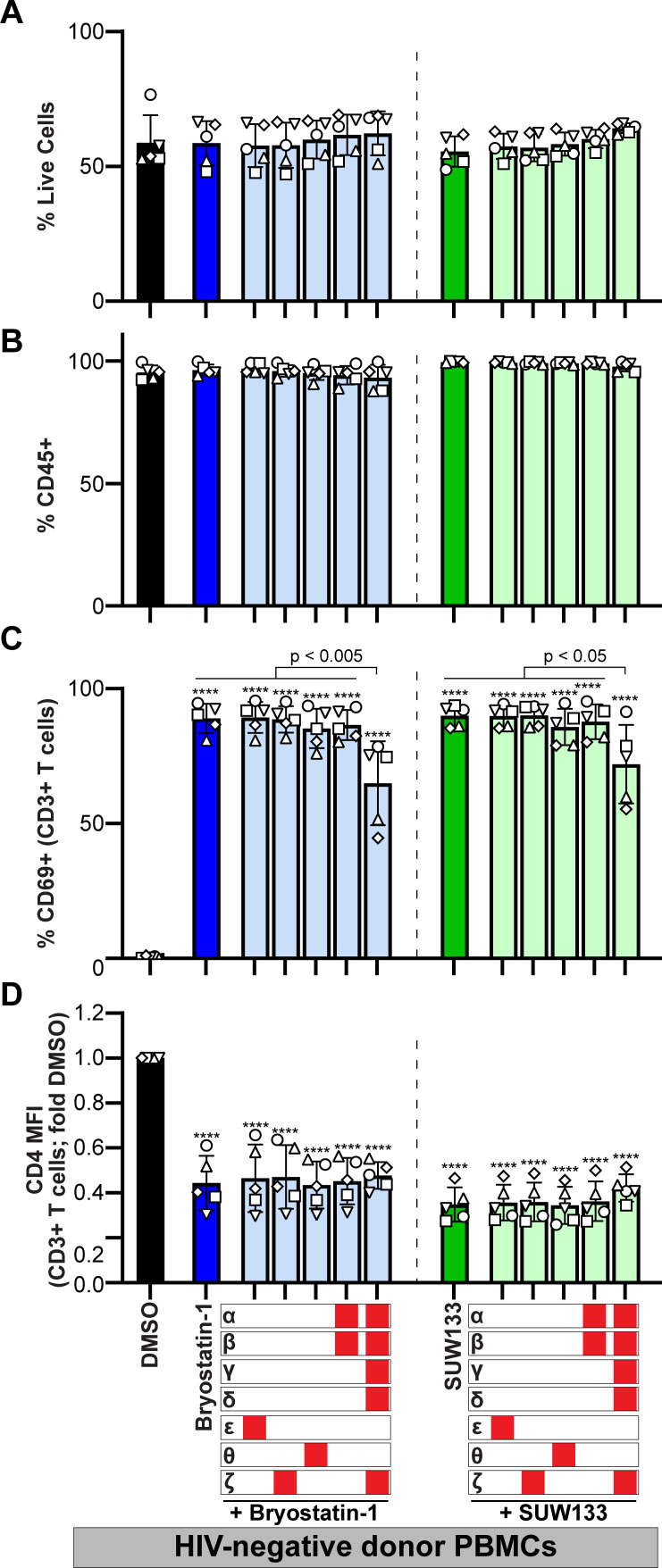
Effects of PKC isoform inhibition on PKC modulator-mediated cell surface immune cell phenotypic changes in HIV-negative PBMCs. PBMCs isolated from HIV-negative donors were pre-incubated with commercially available PKC isoform inhibitors for 3 h and then stimulated with either 20 nM of bryostatin-1 or 10 nM of SUW133 for an additional 21 h (24 h total treatment). Flow cytometry was used to measure the (**A**) percent live cells, (**B**) percent CD45^+^ PBMCs, (**C**) percent CD69^+^ CD3^+^ T cells, and (**D**) relative cell surface CD4 mean fluorescence intensity. The mean and standard deviation are plotted for each condition. *N* = 5 unique HIV-negative donors. Statistical significance was determined using an ordinary one-way ANOVA with a Tukey post hoc test. Conditions that are significantly different from DMSO/solvent control are indicated with asterisks immediately above the bar. Statistically significant differences in conditions are indicated by the grouped *P* value (****P < 0.0001).

Next, we wanted to understand which PKC isoforms were involved in the several reported PKC modulator-mediated immune cell phenotypic alterations, including the cell surface expression of CD69 on CD3^+^ T cells and of CD4 on CD4^+^ T cells. To accomplish this, we treated PBMCs from HIV-negative donors with the same combinations of PKC modulators and isoform inhibitors as were used in the above HIV latency reversal assays. Bulk PBMCs were treated in these experiments as PKC modulators will have immunophenotypic effects on many immune cells (CD4^+^ T cells, CD8^+^ T cells, B cells, and NK cells), which may, independent of LRA activity, affect the tolerability and safety of these compounds. Similar to the results in J-Lat cell lines and in the T_CM_ cell model of HIV latency, there was no significant difference in cell viability among any of the treatments (Fig. 3A). There was also no evidence of a generalized effect on immune cell surface protein expression as no significant difference in the percent of PBMCs expressing CD45/leukocyte common antigen was observed (Fig. 3B). Consistent with previous reports, we observed significant expression of the early T-cell activation marker, CD69, on CD3^+^ T cells by both bryostatin-1 and SUW133 (*P* < 0.0001). Selective inhibition of PKC ε, ζ, θ, or α and β did not inhibit these effects (Fig. 3C). Only pan/broad PKC isoform inhibition (α, β, γ, δ, and ζ) significantly inhibited bryostatin-1-mediated (27% reduction, *P* = 0.0006) and SUW133-mediated (20% reduction, *P* = 0.0062) CD69 upregulation on CD3^+^ T cells (Fig. 3C). Another common immunologic effect of PKC modulators is their ability to potently downmodulate surface expression of CD4 on CD3^+^ T cells. We observed consistent results in PBMCs treated with bryostatin-1 and SUW133 (56% and 65% reduction in surface expression of CD4 on CD3^+^ T cells, respectively; *P* < 0.0001). Interestingly, neither selective nor pan/broad PKC isoform inhibition significantly altered these effects (Fig. 3D).

**Fig 4:**
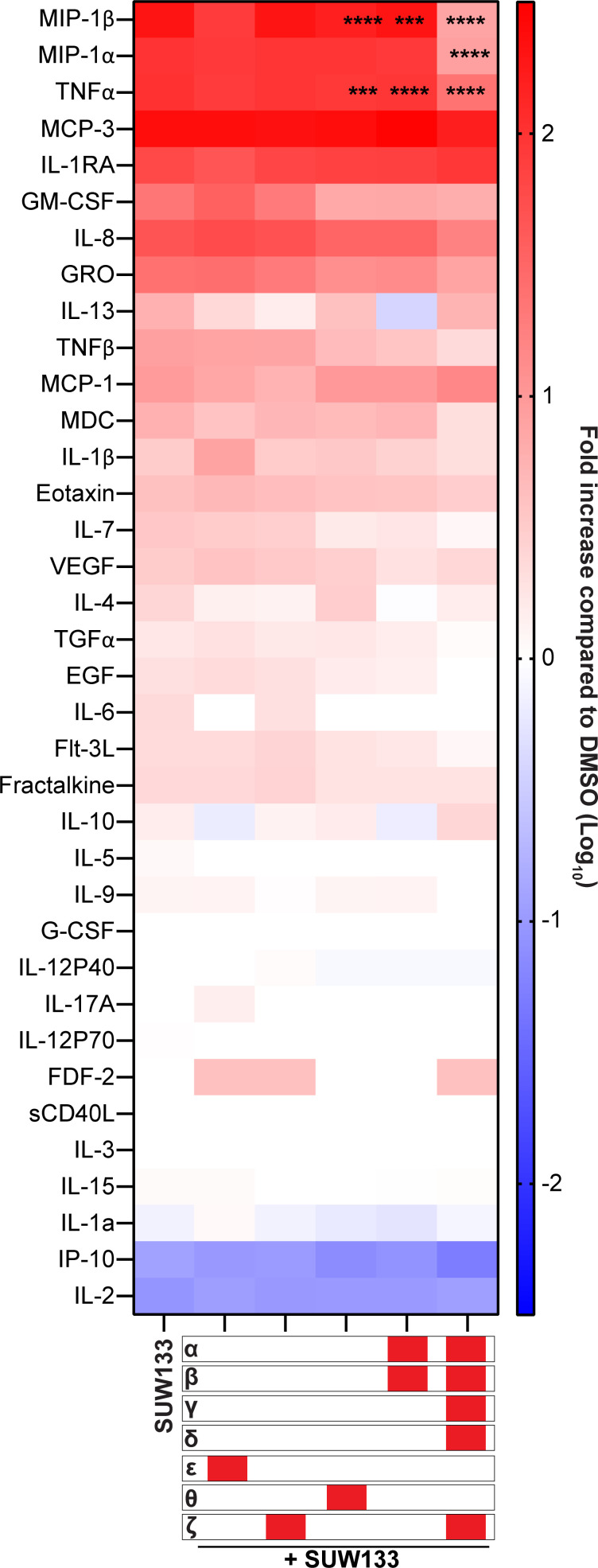
Effects of PKC isoform inhibition on SUW133-mediated cytokine expression from HIV-negative PBMCs. Heatmap summary of relative concentrations of secreted cytokines expressed from treated HIV-negative PBMCs. PBMCs were pre-incubated with commercially available PKC isoform inhibitors for 3 h and then stimulated with 10 nM of SUW133 for an additional 21 h (24 h total treatment). Cytokine levels were normalized for each donor to solvent/DMSO and are expressed as the log_10_ median fold increase. *N* = 3 unique HIV-negative donors. Statistically significant differences between the effects of PKC isoform inhibitors compared to SUW133 treatment alone are shown. Statistical significance was determined using an ordinary two-way ANOVA with a Bonferroni’s post hoc test (***P < 0.001 and ****P < 0.0001).

Finally, we wanted to determine whether PKC isoform inhibition would have any effect on PKC modulator-mediated cytokine production from bulk PBMC cultures. To accomplish this, we treated PBMCs from HIV-negative donors with SUW133 alone or in the presence of the same PKC isoform inhibitors used above and measured the concentrations of cytokines in culture supernatants. Consistent with previous studies, SUW133 elicited potent and broad effects on cytokine production compared to DMSO/solvent control (Fig. 4) (37, 39). Generally, PKC isoform inhibition did not have significant effects on the production of most cytokines. Inhibition of PKC θ or α and β moderately reduced the production of pro-inflammatory cytokines MIP-1β/CCL4 (40–48% reduction, *P* < 0.0006) and TNF-α (48–55% reduction, *P* < 0.0005) (Fig. 4). Broad PKC isoform inhibition (α, β, γ, δ, and ζ) led to a potent reduction in three pro-inflammatory cytokines, including MIP-1β/CCL4 (90% reduction, *P* < 0.0001), MIP-1α/CCL3 (80% reduction, *P* < 0.0001), and TNF-α (91% reduction, *P* < 0.0001) (Fig. 4).

## DISCUSSION

**Fig 5:**
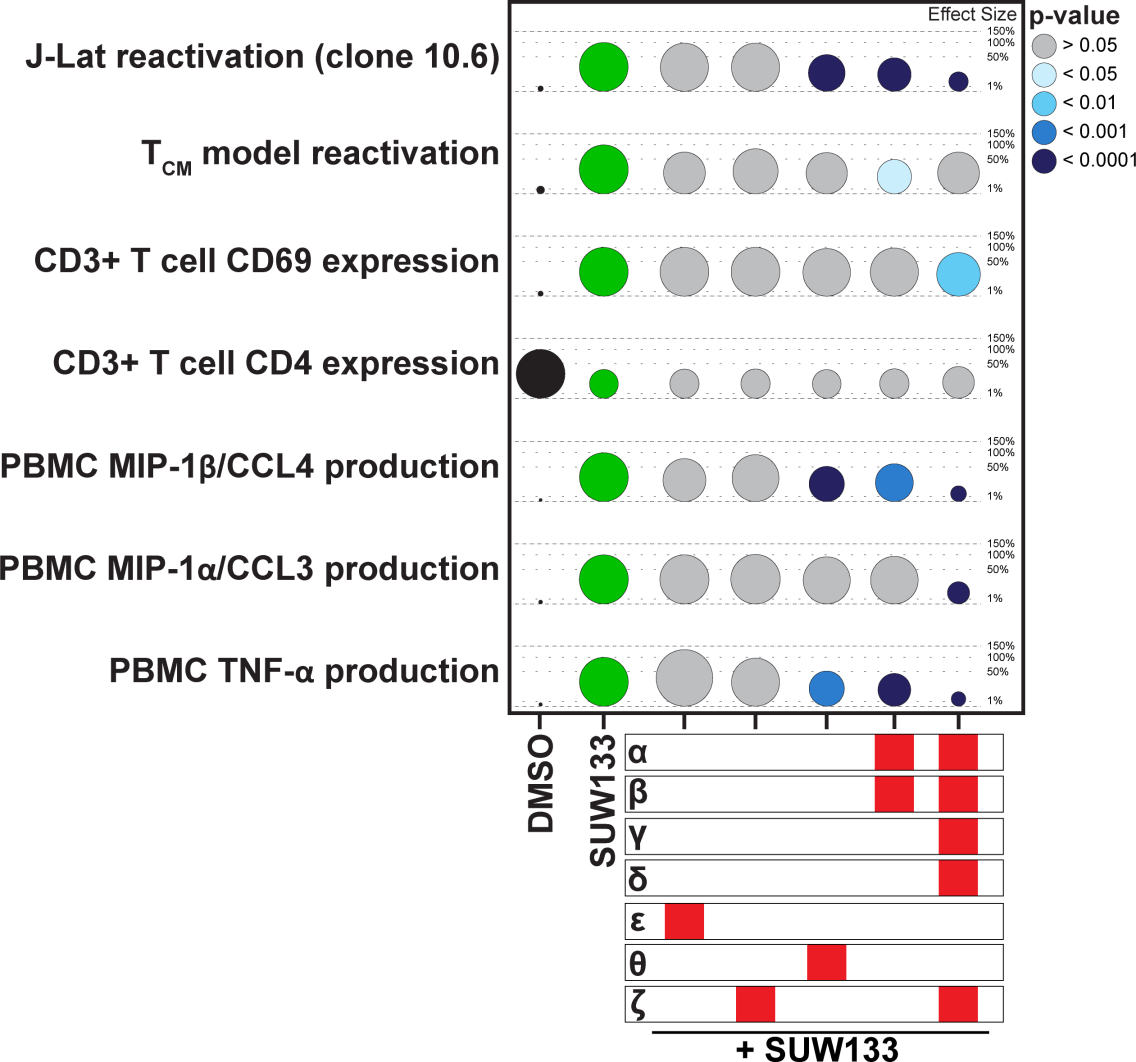
Summary of effects of PKC isoform inhibitors on SUW133-mediated HIV latency reversal and immunomodulation. Balloon plots demonstrating the effect size of treatments on HIV latency reversal, cell surface expression of immune cell markers, and production of pro-inflammatory cytokines/chemokines. Effect sizes for each metric were normalized to SUW133 effect (100%) except CD4 downmodulation, which was normalized to the DMSO/solvent negative control (100%). Statistically significant effects of PKC isoform inhibitors, as compared to SUW133 treatment alone, are indicated by each balloon color.

PKC modulators are among the most potent HIV LRAs available, but clinical translation has been limited by concerns for dose-limiting systemic immune activation and cytokine induction (20, 58). This is likely due to, in part, their non-selectivity for PKC isoforms. Successful clinical application of any LRA regimen will require a systematic preclinical evaluation of LRA effects relevant to downstream clinical translation, such as dosing required for robust latency reversal and possible unacceptable immunomodulatory effects. Figure 5 summarizes the findings from this study and helps to compare the isoforms involved in PKC modulator-mediated effects and their relative contributions. In doing so, PKC α, β, and θ emerge as candidate isoform targets for next-generation selective PKC modulators.

With respect to HIV latency reversal, J-Lat experiments demonstrate that PKC α, β, and θ are necessary for maximal HIV latency reversal by PKC modulators, consistent with previous reports in similar model systems (59). However, using a primary cell model, we demonstrate more subtle effects of these isoforms on HIV latency reversal and much less of an effect by PKC θ. This is somewhat surprising given the critical role of PKC θ in T-cell receptor signaling and activation, including stimulation of downstream pathways thought to be critical for maximum HIV latency reversal such as AP-1, NF-κB, and NFAT (60). Nevertheless, important contributions of the classical isoforms, PKC α and β, to HIV latency reversal were noted in the primary cell assay and suggest a coordination by PKC θ and classical PKC isoforms to release and activate transcription factors (61).

Interestingly, pan-PKC inhibitor treatment did not lead to the greatest reduction of PKC modulator-mediated latency reversal in the primary cell model. In fact, when combined with the more potent PKC modulator tested in this study, SUW133, HIV latency reversal was not significantly reduced below that of SUW133 alone. This highlights the nuanced role of different PKC isoform combinations on latency reversal when they are activated or inhibited in concert. The pan PKC inhibitor used in this study affects PKC α, β, γ, δ, and ζ. The contribution of PKC α and β is discussed above; PKC γ is unlikely to be a factor as it is expressed in neural tissue; and PKC ζ activity does not lead to transactivation of transcription factors critical for HIV latency reversal (62, 63). However, PKC δ negatively regulates T-cell activation and may therefore limit potent HIV latency reversal (64, 65). Similar considerations regarding isoform selectivity for maximal HIV latency reversal have been discussed for other classes of LRAs, including histone deacetylase inhibitors (33).

A recent study described the design and synthesis of a PKC agonist with selectivity for novel PKC isoforms and approximately five- to sixfold specificity for PKC ε over PKC η and θ (66). The authors showed that this novel isoform-selective PKC modulator was capable of *ex vivo* HIV latency reversal from patient-derived cells and avoided toxic side effects in animal models. This work therefore represents an important advance illustrating the benefits of more selective PKC isoform activation in targeted therapeutic applications. However, given the modest selectivity for PKC ε over other novel PKC isoforms, including PKC θ, it cannot entirely be excluded that this compound also acted in part through coordinated agonism of novel PKC isoforms and not only PKC ε. Here, using a pharmacologic inhibitor of PKC ε, we show only a modest, albeit not statistically significant, effect on PKC modulator-mediated HIV latency reversal in a primary cell model. Whether PKC ε agonism, even if only modest, is sufficient for HIV latency reversal to a degree rendering reservoir cells susceptible to clearance, and may, therefore, limit toxicity associated with the activation of other novel PKC isoforms, will be an important consideration in future studies aimed at PKC isoform selectivity.

Immunomodulatory effects of PKC agonists can be both useful and a potential concern for the clinical application of these compounds for HIV cure strategies. Expression of the early T-cell activation marker, CD69, is a reliable marker of bioactivity for PKC modulators (20, 37). Similar to previous reports, we also show that CD69 expression can be affected through PKC-dependent and PKC-independent pathways, with only a modest reduction in PKC modulator-induced CD69 expression even with broad PKC isoform inhibition (67). Importantly, induction of CD69 expression is correlated with the ability of PKC modulators to reverse HIV latency (20, 66). This suggests that factor(s) associated with activated T-cell biology are required for potent HIV latency reversal. Both the CD69 promoter and the HIV long terminal repeat contain binding regions for NF-κB and AP-1, pointing to shared pathways that might explain the utility of CD69 expression as a biomarker of early T-cell activation and PKC modulator-mediated latency reversal (68–70). Another possibility is the activation and activity of P-TEFb, which is required for the efficient transcriptional elongation of HIV and the majority of cellular genes (71, 72). In addition to its apparent correlation with HIV latency reversal, whether transient induction of CD69 by PKC modulators might more broadly affect immune cell function, phenotype, or tissue homing is uncertain (20, 35, 73).

The induction of pro-inflammatory cytokines by SUW133 and other PKC modulators is a potential source of dose-limiting toxicity. Consistent with a previous study, we also found SUW133 to significantly affect a broad range of cytokines and chemokines in bulk PBMC cultures, including the pro-inflammatory molecules MIP-1β/CCL4, MIP-1α/CCL3, and TNF-α (37). Potent induction of these pro-inflammatory cytokines following SUW133 treatment was only significantly reduced by broad/pan-PKC inhibition. Selective PKC isoform inhibition had minimal or inconsistent effects. This suggests that immune activation in response to PKC modulators arises from a coordinated activation of multiple PKC isoforms, indicating a possible approach to partially decouple HIV latency reversal and pro-inflammatory cytokine production through selective PKC agonism.

The clinical toxicity of PKC modulators is still not entirely understood. A recent study has provided some insight into an additional mechanism of toxicity of PKC modulators: platelet activation (66). In their work, the authors demonstrated that the abovementioned newly synthesized PKC agonist with selectivity for novel PKC isoforms and some specificity for PKC ε was able to reverse HIV latency without significant *in vivo* toxicity in animal models by avoiding disseminated intravascular coagulation, a clinical emergency due to diffuse platelet activation. Together with the present study, this work further highlights the potential benefits and feasibility of developing LRAs focused on selective activity against the latent viral reservoir.

Another reproducible result was that PKC isoform inhibition did not prevent PKC modulator-mediated reduction of cell surface CD4 expression on T cells. While we cannot entirely exclude the possibility that other isoforms not directly tested in this study (η, ι/λ, μ) are involved in this process, these data suggest a PKC-independent mechanism of CD4 downmodulation. PKC modulators activate conventional and novel PKC isoforms by binding to the C1 domain (74), which is also found in several other protein families (75). Therefore, it is possible that PKC modulators exert additional PKC-independent effects through other C1 domain-containing proteins. Other proposed PKC-independent effects of bryostatin-1 and related compounds include the protection of infected cells from Chikungunya virus-induced cytopathicity through an, as yet, unknown mechanism; and HIV reactivation through non-C1 domain-containing ERK1/2 MAP kinases (76, 77). This model in which PKC modulators elicit effects in both a PKC-dependent and PKC-independent manner may provide an explanation for any partial decoupling between latency reversal and immunomodulation by PKC isoform inhibitors.

Despite the novel approach to optimizing PKC modulators as LRAs, our study has several limitations. First, while convenient and well-characterized, pharmacologic inhibitors are not entirely isoform specific (78). Thus, future work could focus on genetic approaches to knockdown or knockout specific PKC isoforms or isoform classes. Second, although our primary T_CM_ model is a more relevant HIV latency system than the immortalized J-Lat cell lines, it remains an *in vitro* surrogate for the issue of *in vivo* HIV persistence, where HIV reservoir cells, which are established and affected over the course of years, demonstrate gradual selective pressures and clonal expansion (79). Therefore, extension of this work into patient-derived specimens will be important as future studies look toward the clinical translation of these compounds. Overall, the work presented here addresses a significant objective for future LRA studies, which is the balance between effective HIV latency reversal and the potential for immune-mediated toxicity. By identifying that PKC α, β, and θ are the principal isoforms involved in PKC modulator-mediated latency reversal and that immune activation requires broader and coordinated PKC isoform activity, it may be possible to functionally decouple these effects. More broadly, PKC modulators have shown promise for the treatment of many diseases, including Alzheimer’s disease, multiple sclerosis, and cancer (38, 43, 80). Defining the PKC isoforms that are responsible for the immunomodulatory effects of these compounds, including the induction of specific cytokines and upregulation or downregulation of key immune system proteins, also provides important insights into how these compounds affect immune functions in the context of these other therapeutic applications.

Bryostatin-1 analogs and prodrugs that expand the therapeutic window and alter immunologic signatures have already been developed and described (20, 21, 43, 81, 82). Our data provide critical information for the design of isoform-selective PKC modulators or selective use of PKC inhibitors that maximize HIV latency reversal while minimizing systemic immunotoxicity and allow safe and effective therapy aimed at reducing HIV reservoirs and effecting a cure.

## Data Availability

All data generated during the study are included in this article and associated supplemental material.

## SUPPLEMENTAL MATERIAL

Figure S1 jvi.00500-26-s0001.docx Overview of human primary T_CM_ cell HIV latency model.

ASM does not own the copyrights to Supplemental Material that may be linked to, or accessed through, an article. The authors have granted ASM a non-exclusive, world-wide license to publish the Supplemental Material files. Please contact the corresponding author directly for reuse.
