# Supplementary material for: PKC inhibitors reveal PKC isoforms involved in HIV latency reversal and immunomodulation
Source: J Virol. 2026 Jul 20;100(8):e00500-26. doi: 10.1128/jvi.00500-26 (PMC13483469; doi:10.1128/jvi.00500-26)
Supplement: Supplementary file 1 [file jvi.00500-26-s0001.docx]

Supplemental Figure 1. Overview of human primary T_CM_ cell HIV latency model. (A) Timeline of steps to generate latently infected T_CM_ cells to be used for downstream HIV latency reversal assays. (B) Representative flow plots demonstrating the sort efficiency and purity of the initial naïve CD4+ T cell (T_N_) isolation on day 0 of the assay. (C) Representative flow plots demonstrating the transition of cells in culture to a T_CM_ phenotype by day 7 and at the time of spin inoculation with NL4-3 virus. (D) Representative flow plots demonstrating the initial infection rate in T_CM_ cells with NL4-3 virus (3 dpi) based on intracellular viral gag/p24. (E) Representative flow plots demonstrating the infection rate in T_CM_ cells following 3 days of crowding to promote cell-to-cell spread (6 dpi). (F) Representative flow plots demonstrating the final infection rate (10 dpi; after 4 days of antiretroviral treatment) and the CD4 sort efficiency. CD4+ sorted cells (negative isolation) were then immediately used in HIV latency reversal assay experiments.
